# ANN-Based Airflow Control for an Oscillating Water Column Using Surface Elevation Measurements

**DOI:** 10.3390/s20051352

**Published:** 2020-02-29

**Authors:** Fares M’zoughi, Izaskun Garrido, Aitor J. Garrido, Manuel De La Sen

**Affiliations:** 1 Automatic Control Group-ACG, Department of Automatic Control and Systems Engineering, Faculty of Engineering of Bilbao, Institute of Research and Development of Processes-IIDP, University of the Basque Country-UPV/EHU, Po Rafael Moreno no3, 48013 Bilbao, Spain; izaskun.garrido@ehu.es (I.G.); aitor.garrido@ehu.es (A.J.G.); 2 Automatic Control Group-ACG, Department of Electricity and Electronics, Faculty of Science and Technology, Institute of Research and Development of Processes-IIDP, University of the Basque Country-UPV/EHU, Bo Sarriena s/n, 48080 Leioa, Spain; manuel.delasen@ehu.eus

**Keywords:** acoustic doppler current profiler, airflow control, artificial neural network, oscillating water column, power generation, stalling behavior, wave energy, Wells turbine

## Abstract

Oscillating water column (OWC) plants face power generation limitations due to the stalling phenomenon. This behavior can be avoided by an airflow control strategy that can anticipate the incoming peak waves and reduce its airflow velocity within the turbine duct. In this sense, this work aims to use the power of artificial neural networks (ANN) to recognize the different incoming waves in order to distinguish the strong waves that provoke the stalling behavior and generate a suitable airflow speed reference for the airflow control scheme. The ANN is, therefore, trained using real surface elevation measurements of the waves. The ANN-based airflow control will control an air valve in the capture chamber to adjust the airflow speed as required. A comparative study has been carried out to compare the ANN-based airflow control to the uncontrolled OWC system in different sea conditions. Also, another study has been carried out using real measured wave input data and generated power of the NEREIDA wave power plant. Results show the effectiveness of the proposed ANN airflow control against the uncontrolled case ensuring power generation improvement.

## 1. Introduction

Ocean energy is a more advantageous renewable resource compared to solar and wind. It is more reliable since it is consistent throughout the day and night and more predictable, which can be foreseen several days in advance, and has significantly denser energy. Oceans represent two-thirds of the earth’s surface and store the energy emitted by the sun, making up the largest source of renewable energy. The energy within may be harnessed in various forms, such as ocean waves, ocean currents, tidal range, tidal currents, ocean thermal energy, and salinity gradients [1,2]. However, despite all of these points, these industries are still underdeveloped [3].

Many ocean energy technologies have emerged to exploit this resource, especially for tidal and wave conversion systems. Both technologies are predicted to contribute the most to the European energy platform in the short to medium term (2025–2030) [4,5]. However, the majority of ocean energy-related industries are still in the early stage of development, ranging from theory, design, up to the demonstration phases [6]. In wave energy conversion, converters of various types can be found across Europe, including oscillating water columns like Limpet in Scotland and NEREIDA in Spain [7], point absorber buoys like PowerBuoy in the UK [8], surface attenuators like the Pelamis in Portugal [9], overtopping devices like the Wave Dragon in Denmark [10], converters for tidal energy conversion, including horizontal axis turbines like SeaGen in Ireland [11], vertical axis turbines like Kobold in Italy [12], and variable foil turbines like the Stingray in the UK [13].

Energy hoarded in waves is the form of ocean energy with the highest deployment potential in European waters. It is known that the waves have a global energy potential 30 times higher than that of tides [5]. Moreover, the latest progress of developed wave energy converters (WEC) and projects offered favorable outcomes, which advanced the wave industry to competitive ranks. These factors attracted the attention of investors and policymakers to wave energy; this has been noticed in some countries that started to assess the abundant resource for possible projects, including Hawaii, Italy, India, Peru, and many others [14,15,16,17]. For countries with advanced projects, efforts were invested in the deployment and integration of full-scale converters, which led some technologies to break into commercial implementations [7,18,19]. By slowly earning more and more confidence from investors and efforts to help research and development of ocean energy, industries started to increase. In fact, the EU-funded related research themes where 68% of the funds were invested in technology development divided into 45% for wave technology development and 23% for tidal technology development [20]. Nowadays, new developments are aiming to maintain this confidence by focusing on reliability, robustness, and reduction of costs and risks [21].

One of the most employed WECs is the oscillating water column (OWC) for its simplicity and feasibility. However, OWCs with Wells turbine-based PTO systems suffer from the stalling effect, which hinders the turbine’s efficiency, leading to a decrease in the energy extraction and limitation of the generated power [22,23]. This paper presents the modeling and control of an OWC system. The proposed control strategy aims to help the PTO avoid the power limitation from the stalling effect. In this context, a novel ANN-based airflow control has been designed and implemented with the objective of controlling an air valve inside the capture chamber of the OWC. This will allow the regulation of the airflow velocity below the critical point that provokes the stalling behavior by considering the wave input characteristics. The ANN has been trained using real wave data measured at the coast of Mutriku using an acoustic doppler current profiler (ADCP), and its capabilities were adopted to recognize the incoming peak waves causing the stalling behavior and provide the adequate reference value of the desired airflow speed for the airflow control scheme.

An airflow control strategy has been proposed to OWC converters to control the airflow rate through the turbine, which should be prevented from exceeding the threshold value beyond which severe internal aerodynamic blade stalling reduces the power output. This is achieved by using a throttle valve (in series) or a by-pass valve (in parallel), as suggested by Falcão et al. in [24]. This strategy has been further implemented using various control approaches, such as the PID controllers used by Amundarain et al. [25], and Mishra et al. improved the control scheme by using fractional order PID (FOPID) in [26], which offered to the controller more flexibility in regular and irregular waves. Later intelligent tuning techniques were introduced by M’zoughi et al. using advanced metaheuristic optimization algorithms and fuzzy gain scheduling (FGS) in [27].

Artificial neural networks are able to efficiently approximate and interpolate multivariate data that would require massive databases [28]. The ANN approach is acknowledged for nonlinear statistical fitting applications [29,30]. Automatic control applications and control design applications have adopted ANN in many nonlinearity problems [31,32,33]. The nonlinearity aspect and complexity of the oscillating water column system makes the ANN a very promising solution to deal with the stalling behavior problem.

Recently, the world has generated electric power from wind turbines, but the airflow control strategy for wind turbines has uncertainty. However, with the developments in meteorological temperature and humidity predictions from Fourier-statistical analysis of hourly data, which guarantees its operation [34,35,36], the temperature readings for all the 365 days and the 24 h may provide a strong stochastical connection to the classic strategy. Likewise, the neural network models were able to perform cold wind control [37,38,39]. Consequently, knowledge through measurements of climatic variables is the amount of airflow received per unit area, which explains the weather records used to estimate airflow on wind turbines available at a certain point on the surface of the earth. Therefore, the best option for a perpetual energy solution is obtaining electricity from mechanical energy generated by the movement of sea waves [40,41,42].

The rest of this paper is organized as follows; Section 2 presents the model statement by mathematically describing the different parts of the OWC plant. The problem statement is presented in Section 3 by introducing the stalling behavior and its effect on the produced torque. Section 4 explains the proposed ANN-based airflow control and the designed ANN reference generator. Section 5 sets a demonstrative study case to study the effectiveness of the proposed ANN-airflow control in different wave conditions. Finally, Section 6 finishes the article with some concluding remarks.

## 2. Model Statement

**Figure 1:**
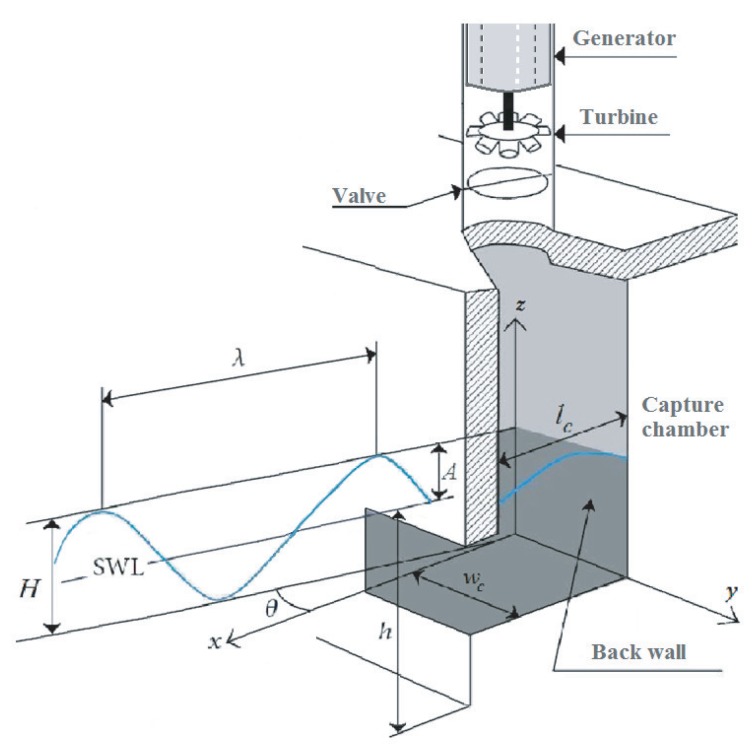
Scheme of an oscillating water column system and the sea wave.

This section presents the modeling of the parts of the wave energy converter under study, which is the oscillating water column shown in Figure 1. This includes the mathematical models of the waves, capture chamber, Wells turbine, and the generator.

### 2.1. Wave Surface Dynamics

In this article, monochromatic unidirectional waves are considered as the input to the numerical model of the plant. To describe its surface dynamics, various wave theories may be used, such as Cnoidal wave theory, second or higher-order Stokes theory, and Airy linear theory [43,44].

The simplest description of ocean waves and the most widely used is the Airy wave theory. It expresses the waves as ideal sinusoidal waves by neglecting turbulence, friction losses, and other energy losses [44].

The outline of an ocean wave is illustrated in Figure 1, where SWL is the still water level, *h* is the sea depth from the seabed to SWL. *H* is wave height from the wave trough to the wave crest. *A* is the wave amplitude from SWL to the wave crest, and λ is the wavelength, which is the distance between successive crests [44,45].

Hence, the surface elevation of an ocean wave may be written as [46,47]:

(1) $$z\left(x,t\right)=A\sin\left(\omega t-kx\theta \right)=H/2\sin\left(\omega t-kx\theta \right)$$

where *x* is a horizontal coordinate in which the positive direction is the direction of wave propagation, with x=0 at the back wall, θ is the angle between the *x*-axis and the direction of wave advance, and *k* is the wave number, which is related to ω by the dispersion relationship of Equation (2) defined in [46].

(2) $$k\tanh\left(kh\right)=\omega^{2}/g$$

where ω is the wave frequency, and *g* is the acceleration gravity.

### 2.2. Capture Chamber Model

The air volume in the chamber of an OWC is given in [48,49,50] as:

(3) $$V\left(t\right)=V_{c}+\frac{w_{c}H}{k}\sin\left(kl_{c}/2\right)\sin\left(\omega t\right)$$

where $V_{c}$, $w_{c}$, and $l_{c}$ are the capture chamber’s volume, inner width, and length, respectively.

From Equation (3), the volume flow rate may be described as [48,49,50]:

(4) $$Q\left(t\right)=w_{c}cH\sin\left(\frac{kl_{c}}{2}\right)\cos\left(\omega t\right)$$

with $c=w_{c}/k$.

By using Equation (4) and taking into consideration the topology of the capture chamber, the axial airflow speed may be defined as [48,49,50]:

(5) $$v_{x}\left(t\right)=\frac{Q(t)}{S}=\frac{8Acw_{c}}{\pi D^{2}}\sin\left(\frac{\pi l_{c}}{cT_{w}}\right)\cos\left(\frac{2\pi }{T_{w}}t\right)$$

where *D* is the duct diameter.

### 2.3. Wells Turbine Model

**Figure 2:**
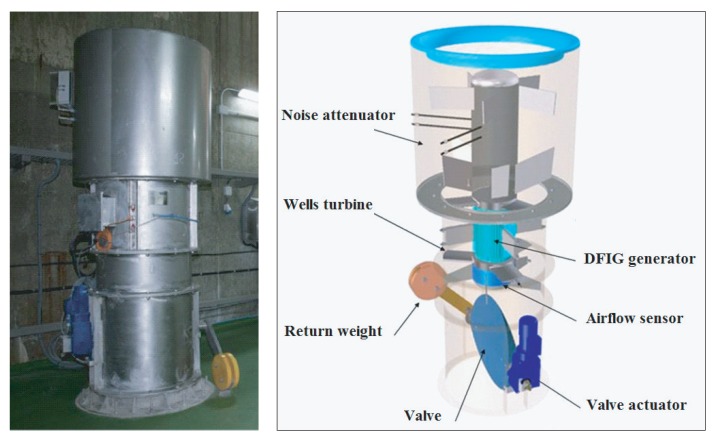
PTO of an oscillating water column (OWC) combining Wells turbine and doubly fed induction generator (DFIG).

The turbine under study, shown in Figure 2, was invented by Dr. Allan Wells in the mid-1970s [47,51] and is a self-rectifying axial-flow turbine, which provides a unidirectional rotational movement regardless of the airflow direction thanks to its special geometry [52,53].

The Wells turbine may be described by the equations given in [23]:

(6) dp=CaK11aavx2+rωr2

(7) $$\begin{array}{ccc} K & = & \rho lbn/2 \end{array}$$

(8) $$\begin{array}{ccc} T_{t} & = & rC_{t}K\left[v_{x}^{2}+{\left(r\omega_{r}\right)}^{2}\right] \end{array}$$

(9) $$\begin{array}{ccc} \phi & = & v_{x}{\left(r\omega_{r}\right)}^{-1} \end{array}$$

(10) $$\begin{array}{ccc} Q & = & av_{x} \end{array}$$

(11) $$\begin{array}{ccc} \eta_{t} & = & T_{t}\omega_{r}{\left(dpQ\right)}^{-1}=C_{t}{\left(C_{a}\phi \right)}^{-1} \end{array}$$

where dp is the pressure drop, $C_{a}$ and $C_{t}$ are, respectively, the power and torque coefficients, ϕ is the flow coefficient, $T_{t}$, $\eta_{t}$, *K*, *r* are, respectively, the turbine’s torque, performance, constant, and mean radius, *l*, *b*, *n* are, respectively, the blade’s chord length, height, and number, $\omega_{r}$ is the angular velocity, *a* is the cross-sectional area, and ρ is the air density.

**Figure 3:**
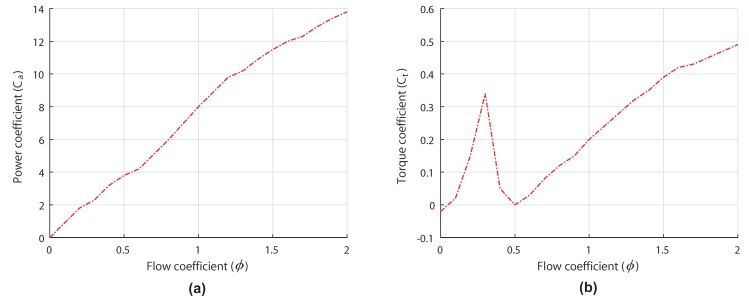
Characteristic curves of the installed Wells turbine [27]. (**a**) Power coefficient versus flow coefficient. (**b**) Torque coefficient versus flow coefficient.

The curves of both the power coefficient $C_{a}$ and the torque coefficient $C_{t}$ against the flow coefficient ϕ form the characteristic curves of the studied Wells turbine, which are presented in Figure 3.

### 2.4. Doubly Fed Induction Generator Model

The DFIG model may be described by the equations given in [54,55,56].

The voltages across the stator and rotor in the dq frame may be written as [54,55,56]:

(12) $$\begin{array}{c} \left\{\begin{array}{c} v_{ds}=R_{s}i_{ds}+\frac{d\psi_{ds}}{dt}-\omega_{s}\psi_{qs}\\ v_{qs}=R_{s}i_{qs}+\frac{d\psi_{qs}}{dt}+\omega_{s}\psi_{ds} \end{array}\right. \end{array}$$

(13) $$\begin{array}{c} \left\{\begin{array}{c} v_{dr}=R_{r}i_{dr}+\frac{d\psi_{dr}}{dt}-\omega_{r}\psi_{qr}\\ v_{qr}=R_{r}i_{qr}+\frac{d\psi_{qr}}{dt}+\omega_{r}\psi_{dr} \end{array}\right. \end{array}$$

where $R_{s}$ and $R_{r}$ are the stator and the rotor resistances, $\omega_{s}$ and $\omega_{r}$ are the stator and the rotor angular velocities, $i_{ds}$ and $i_{qs}$ are the *d*-*q* stator currents, $i_{dr}$ and $i_{qr}$ are the *d*-*q* rotor currents.

The flux linkage in the stator and the rotor may be written as:

(14) $$\begin{array}{c} \left\{\begin{array}{c} \psi_{ds}=L_{ss}i_{ds}+L_{m}i_{dr}\\ \psi_{qs}=L_{ss}i_{qs}+L_{m}i_{qr} \end{array}\right. \end{array}$$

(15) $$\begin{array}{c} \left\{\begin{array}{c} \psi_{dr}=L_{rr}i_{dr}+L_{m}i_{ds}\\ \psi_{qr}=L_{rr}i_{qr}+L_{m}i_{qs} \end{array}\right. \end{array}$$

where $L_{ss}$ and $L_{rr}$ are the stator and the rotor inductances, and $L_{m}$ is the magnetizing inductance.

The generated electromagnetic torque may be expressed as:

(16) $$T_{e}=\frac{3}{2}p\left(\psi_{ds}i_{qs}-\psi_{qs}i_{ds}\right)$$

where *p* is the pair pole number.

The mechanical interaction between the turbine and the generator can be defined as:

(17) $$\frac{J}{p}\frac{d\omega_{r}}{dt}=T_{e}-T_{t}$$

where *J* is the inertia of the system.

## 3. Problem Statement

The OWC understudy consists of a Wells turbine coupled to DFIG, however, as already mentioned, the aerodynamics of the turbine gets affected by the stalling phenomenon when reaching a critical flow rate value. In fact, this behavior occurs when the airflow velocity $v_{x}$ rises quickly, but the slow dynamics of the DFIG prevent the rotational speed $\omega_{r}$ from changing as quick. The stalling effect can be deduced from Figure 3b, which indicates that when the flow coefficient ϕ surpasses a certain threshold (which is 0.3 for this turbine), the torque coefficient $C_{t}$ drops drastically.

**Figure 4:**
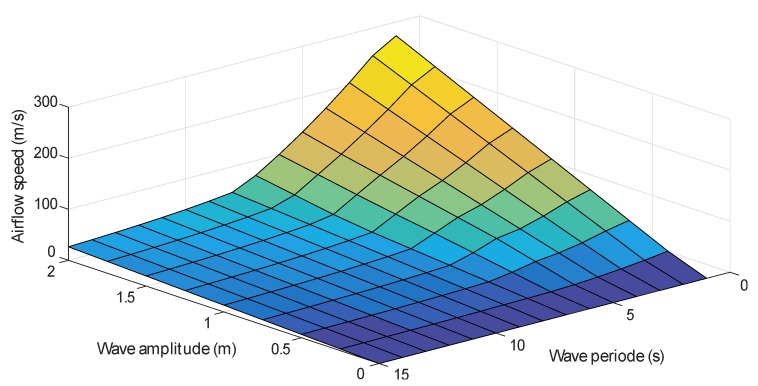
Airflow speed variation for different wave amplitudes and periods.

The flow coefficient ϕ described by Equation (9) depends on the airflow speed $v_{x}$. However, as described by Equation (5), $v_{x}$ depends on the wave amplitude and period. In fact, the higher the wave amplitude and the shorter the period are, the stronger the wave power and the airflow speed are, as explained by Figure 4.

**Figure 5:**
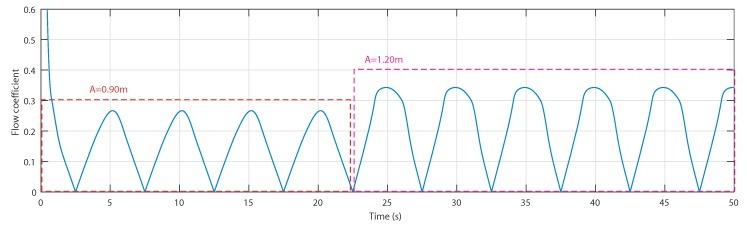
Flow coefficient vs. time for two wave amplitudes [27].

The stalling effect is shown by studying the uncontrolled OWC plant in two sea conditions; the first one is a wave with a 10 s period and a wave amplitude of 0.9 m starting from 0 s to 22.5 s and the second one is a wave with a 10 s period and a wave amplitude of 1.2 m from 22.5 to 50 s. Figure 5 shows that the first sea condition provides a flow coefficient lower than the threshold value 0.3, whereas, with the second sea condition, it exceeds the threshold value.

**Figure 6:**
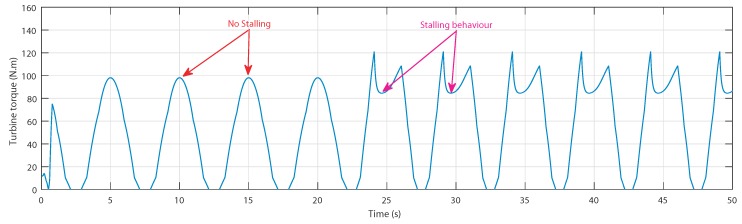
Turbine torques vs. time for two sea conditions [27].

Accordingly, the obtained torques shown in Figure 6 indicate no stalling for the first sea condition and a drastic decrease at the crest in the second sea condition by the stalling effect. This will decrease the produced torque in terms of the average value.

## 4. Control Statement

**Figure 7:**
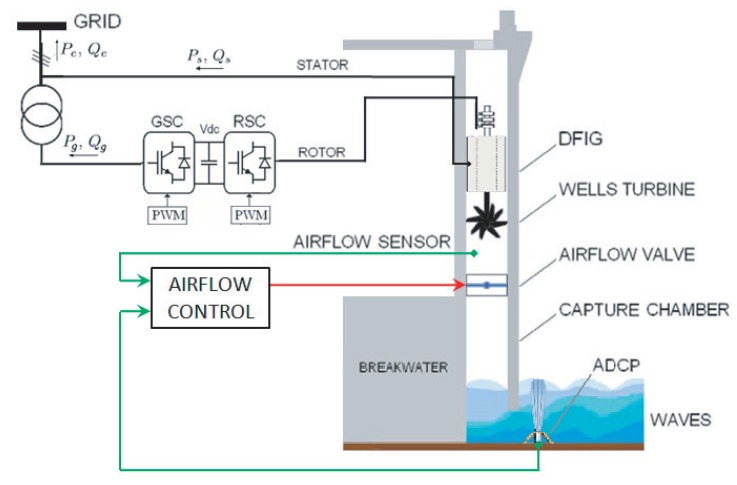
Airflow control scheme for a Wells turbine-based OWC wave power plant.

The stalling phenomenon of the Wells turbine may be avoided by continuously adjusting the flow coefficient [48,49]. By referring to Equation (7), the flow coefficient depends on the airflow speed in the turbine duct. Hence, regulating the airflow velocity $v_{x}$ will help avoid the stalling effect. In this sense, an ANN-based airflow control scheme has been proposed to control the air valve situated inside the capture chamber of the OWC system in order to regulate the airflow speed $v_{x}$, thus keeping the flow coefficient ϕ under the threshold value. The ANN will be trained to recognize incoming waves from measured data by the acoustic doppler current profiler (ADCP). The scheme of the proposed ANN-airflow control strategy is presented in Figure 7.

### 4.1. ANN-Based Airflow Control

The capture chambers in NEREIDA are equipped with air valves at the summit connecting to the turbine duct. The valve is mounted in series with the Wells turbine, followed by the DFIG, as illustrated in Figure 7. It separates the air and pressure in the chamber from that of the capture chamber, and its role is to prevent the pressure difference from surpassing its critical limit [50]. However, this device is also useful in controlling the amount of airflow input. The valve is operated through its actuator, which turns the valve plate to the desired angular position and then holds it by an electromagnetic brake. The flow rate will vary depending on the degree of the opening of the valve plate.

**Figure 8:**
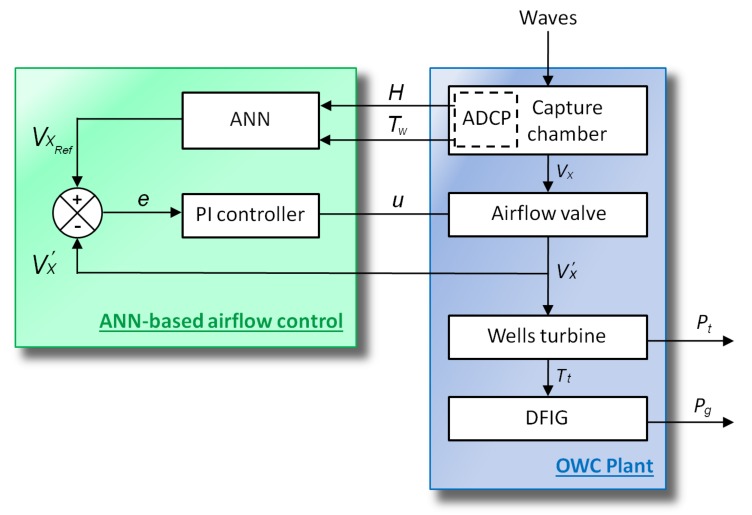
Artificial neural networks (ANN)-based airflow control block diagram for a Wells turbine-based OWC.

This control strategy aims to control the air valve using a proportional integral (PI) controller, as explained by the block diagram of Figure 8. This is achieved by measuring the airflow velocity in the turbine duct $V_{X}^{\prime }$ and comparing it to the reference velocity $V_{XRef}$. The reference velocity $V_{XRef}$ is provided by the ANN airflow velocity reference generator. The output of the PI controller is the control signal for the valve actuator.

**Figure 9:**
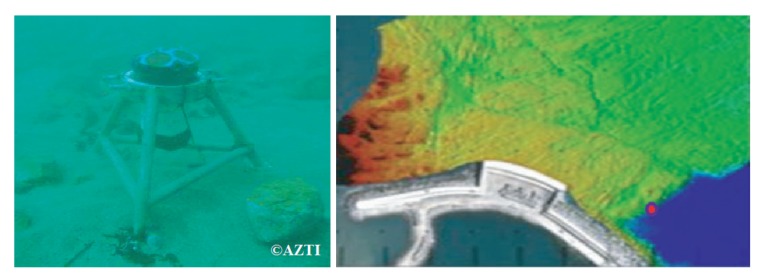
Acoustic doppler current profiler in Mutriku.

The ANN will be trained to recognize incoming waves from measured data gathered by AZTI-Tecnalia at the breakwater of Mutriku in the northern coast of Spain. Both inputs of the designed ANN are obtained from the acoustic doppler current profiler (ADCP) installed at Mutriku (shown in Figure 9). The considered data are from 12 May 2014, where measurements of the first 20 min were recorded every 2 h. The installed ADCP is of Workhorse 600 kHz from Teledyne RDI with a sampling period of 0.5 s.

ADCPs are hydro-acoustic devices used to measure water current velocities. This technology uses the Doppler effect of sound waves scattered back from particles. It is able to send and receive sound signals thanks to piezoelectric transducers. The time it takes sound waves to travel allows estimating the distance. The frequency shift of the echo is proportional to the water velocity within the acoustic path. Three-dimensional velocity measurements require a minimum of three beams [57,58].

In the last few years, more functionality has been added to ADCPs to include wave and turbulence measurements. The ADCP device specific to wave characteristic measurements is known as the acoustic wave and current (AWAC) profiler, which allows the measurement of surface wave height and its direction [59,60]. The one installed in Mutriku is mounted upwards down at the seafloor. The AWAC device has four transducers that transmit four beams; one transducer in the middle point vertically up, and three transducers symmetrically positioned at 120 degrees from each other and surrounding the center one, and they are angled off the vertical by 25 degrees [61,62].

### 4.2. ANN Airflow Velocity Reference Generator

**Figure 10:**
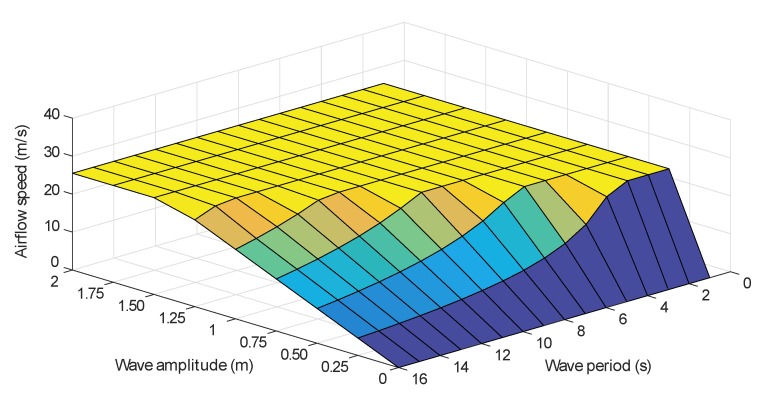
Desired airflow speed for different wave amplitudes and periods.

The trained ANN has to generate reference airflow velocity that does not provoke the stalling behavior of the Wells turbine, this means that when the wave power is low, the total airflow is admitted by the valve, but when the waves are strong enough to generate high airflow speed, the valve should block the incoming airflow to reduce its speed. In this sense, numerous simulations were carried out to record the acceptable waves and distinguish the strong waves that provoke the stalling effect. Hence, input–output data sets have been assembled to train the network into generating the desired output. The desired airflow reference as a function of the wave amplitude and period is shown in Figure 10.

The ANN developed is a feed-forward network controller that consists of an input layer of two neurons; one for the wave amplitude, and the other one for the wave period, and an output layer with one neuron representing the desired airflow speed reference. Determining the number of hidden layers/neurons in such a complex system is an intricate task; therefore, the trial-and-error rule based on the forward approach procedure has been used [63]. The method begins with an undersized number of hidden layers/neurons and then increases them. At every time, the new network structure is trained and tested. This process is repeated until the train and test results are improved. By statistical analysis, the best structure is selected by choosing the best mean squared error (MSE).

ANNs consist of neurons in the input, output, and hidden layers, which are connected with weighted signals ($w_{ij}$). The neurons consist of a bias $b_{j}$, a sum function $S_{j}$ coupled to an activation function $\varphi_{j}$. The sum function may be defined as:

(18) $$S_{j}=\sum_{j=1}^{N}\left(w_{ji}y_{i}\right)+b_{j}$$

where $S_{j}$ is the sum from the $j^{th}$ neuron from the current layer, $b_{j}$ is the bias, *N* is the total number of neurons, $w_{ji}$ is the weight of the signal connecting the $j^{th}$ neuron from the current layer with the $i^{th}$ neuron from the previous layer, and $y_{i}$ is the input signal from the neuron of the previous layer.

The activation function of neurons in the input layer is clamped by the input vector; however, the activation function of the rest of the neurons in the network can be chosen as:

(19) $$y_{j}=\varphi_{j}\left(S_{j}\right)=\left\{\begin{array}{c} S_{j}\;\;\;\;\left(Linear\right)\\ \frac{1}{1+e^{-S_{j}}}\;\;\left(Sigmoid\right)\\ \frac{e^{S_{j}}-e^{-S_{j}}}{e^{S_{j}}+e^{-S_{j}}}\;\left(Hyperbolic\;tangent\right) \end{array}\right.$$

To train the ANN, the Levenberg–Marquardt (LM) algorithm has been considered as a learning method [64]. This algorithm is a variant of Newton’s method, which was designed to minimize functions that are sums of squares of nonlinear functions [65]. The algorithm aims to update the network weights in order to minimize the performance index by [66]:

(20) $$∆X={\left[\nabla^{2}F\left(X\right)\right]}^{-1}\nabla F\left(X\right)$$

where F(X) is the performance index, ∇F(X) is the gradient, and $\nabla^{2}F\left(X\right)$ the Hessian matrix.

Knowing that the performance index is defined as:

(21) $$F\left(X\right)=\sum_{i=1}^{N}E_{i}^{2}\left(X\right)=E^{T}\left(X\right).E\left(X\right)$$

where E(X) is the error between the ANN’s output and the target output.

The gradient of the performance index may be written as:

(22) $$\nabla F\left(X\right)=2J^{T}\left(X\right).E\left(X\right)$$

where J(X) is the Jacobian matrix defined as [67]:

(23) $$J\left(X\right)=\left(\begin{array}{cccc} \frac{\partial E_{1}\left(X\right)}{\partial X_{1}} & \frac{\partial E_{1}\left(X\right)}{\partial X_{2}} & \ldots & \frac{\partial E_{1}\left(X\right)}{\partial X_{n}}\\ \frac{\partial E_{2}\left(X\right)}{\partial X_{1}} & \frac{\partial E_{2}\left(X\right)}{\partial X_{2}} & \ldots & \frac{\partial E_{2}\left(X\right)}{\partial X_{n}}\\ \vdots & \vdots & \ddots & \vdots \\ \frac{\partial E_{N}\left(X\right)}{\partial X_{1}} & \frac{\partial E_{N}\left(X\right)}{\partial X_{2}} & \cdots & \frac{\partial E_{N}\left(X\right)}{\partial X_{n}} \end{array}\right)$$

where *n* is the number of training patterns.

Hence, the Hessian matrix may be expressed as:

(24) $$\nabla^{2}F\left(X\right)=2J^{T}\left(X\right).J\left(X\right)+2\sum_{i=1}^{N}E_{i}\left(X\right).\nabla^{2}E_{i}\left(X\right)$$

## 5. Results and Discussion

This section presents the simulations carried out to evaluate the performance of the proposed ANN-based airflow control. To evaluate the performance of the proposed control methodology, the OWC wave power plant has been implemented by a complete wave-to-wire model on Matlab/Simulink, and a comparison between the uncontrolled and controlled cases has been performed.

To implement the OWC model, we considered the parameters obtained from real measurements at the NEREIDA wave power plant. These parameters were provided by the Basque Energy Agency (EVE) and are detailed in Table 1.

### 5.1. ANN Training Performance

Using the trial-and-error rule based on the forward approach procedure, several structures with different hidden layers and neurons were tested with our problem in order to obtain the best MSE. The activation function used for the neurons in the hidden layer is the hyperbolic tangent, whereas in the output layer, the linear activation function. During the simulation tests, the comparative study takes into account the number of epochs and the least MSE. The training process was performed on computers with a 3.2 GHz Intel Core i7-8700 Six-Core processor and Integrated Intel UHD Graphics 630. Table 2 shows the results obtained of training different networks with different structures applied to the problem of wave characteristics recognition.

From the results of Table 2, we can deduce that the results obtained using a single hidden layer networks, known as Vanilla neural networks, performs poorly due to the nonlinearity and complexity of the problem. On the other hand, network structures with more than one hidden layer, known as deep neural networks, offer better performance. Therefore, further analysis was carried out on the curves of the training performance of the deep neural network structures, with the best results marked as ANN1 (2 × 2 × 4 × 1) and ANN2 (2 × 4 × 4 × 1) in order to select the adequate structure.

**Figure 11:**
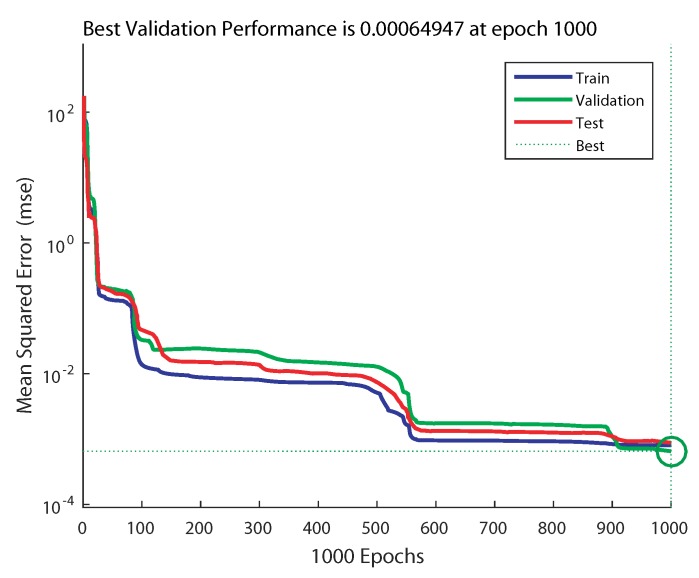
Training performance for a network of 2 × 2 × 4 × 1 (ANN1).

**Figure 12:**
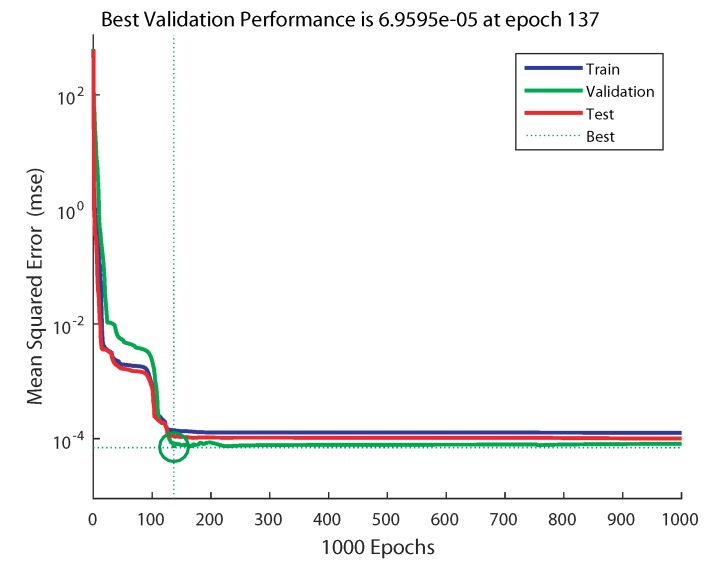
Training performance for a network of 2 × 4 × 4 × 1 (ANN2).

The evolution of the training process of ANN1 (2 × 2 × 4 × 1) is depicted in Figure 11, and the one of ANN2 (2 × 4 × 4 × 1) is depicted in Figure 12. From these figures, it can be noticed that in the case of ANN1, the training process took more epochs to converge to the minimum MSE of 6.4947 × 10${}^{-4}$; however, with ANN2, it converged to a smaller MSE of 6.9595 × 10${}^{-5}$ in fewer epochs at 137.

Finally, the topology of ANN2 has been selected as the ANN airflow velocity reference generator to be implemented in the proposed airflow control scheme to provide the desired airflow speed.

### 5.2. Control Assessment with Regular Waves

**Figure 13:**
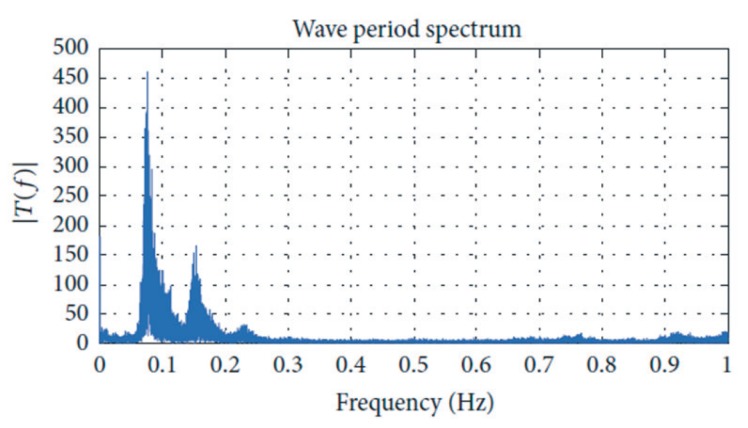
Representative spectral analysis of the waves at the site Mutriku on 12 May 2014 [27].

To evaluate the power generation enhancement of the proposed ANN-based airflow control scheme, a study case considering two representative sea states from the site of Mutriku are selected from the wave spectrum of the location illustrated in Figure 13.

**Figure 14:**
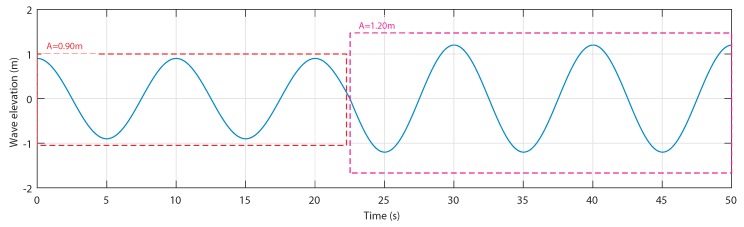
Considered wave input to the implemented OWC model.

The waves are characterized by a water depth h= 5 m and a wave period $T_{w}=10$ s. The first wave amplitude is A=0.9 m from 0 s to 22.5 s, and the second wave amplitude is A=1.2 m from 22.5 s to 50 s, as shown in Figure 14.

**Figure 15:**
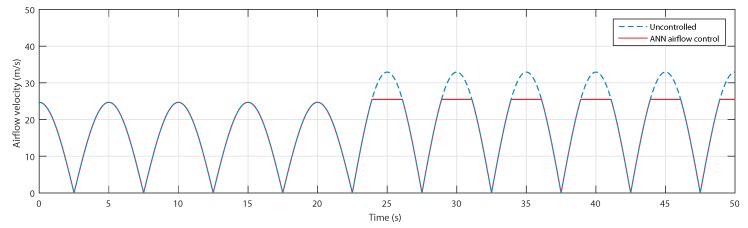
Airflow speed for uncontrolled case and ANN-based airflow control.

Figure 15 shows the airflow speed for both waves in the uncontrolled and controlled cases. The curves show that for the first sea condition, the airflow velocity is in the acceptable range, whereas for the second wave, the neural network recognized the wave as one that will provoke a stalling behavior in the OWC; therefore, the control has been activated and the speed has been reduced by the air valve.

**Figure 16:**
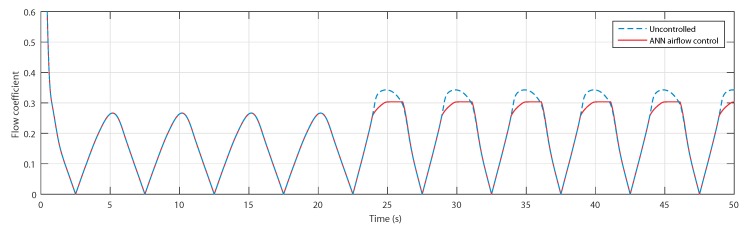
Flow coefficients for uncontrolled case and ANN-based airflow control.

The flow coefficients are presented in Figure 16 and show that, indeed, the flow coefficient of the first wave did not exceed the threshold value of 0.3 in the uncontrolled case, which required no action in the controlled case. However, in the case of the second wave, the flow coefficient did exceed the threshold value; hence, the control managed to successfully adjust it.

**Figure 17:**
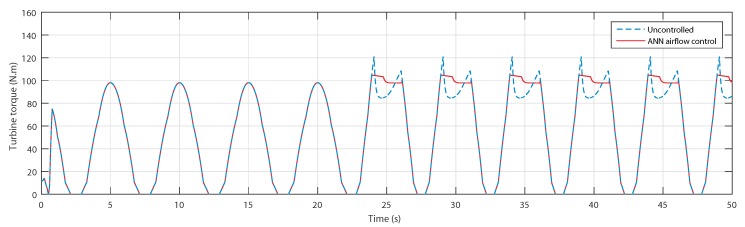
Turbine torque for uncontrolled case and ANN-based airflow control.

The resulting produced torques are shown in Figure 17, where it is clearly observed that with the second wave, the torque in the uncontrolled case has a drastic decrease at every crest, which reduces the torque in terms of average value. However, using the ANN-airflow control, the torque has been maintained at the maximum value, thus maintaining a higher average value.

**Figure 18:**
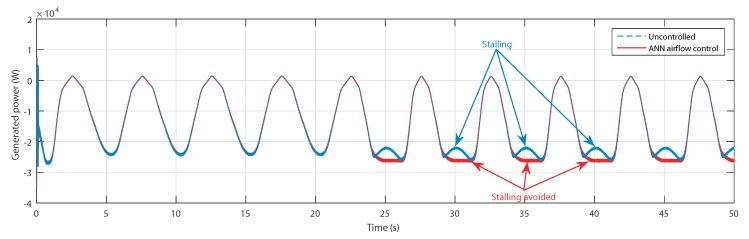
Generated power for uncontrolled case and ANN-based airflow control.

Eventually, the generated power avoided the power drop at the peak of the second wave, as shown in Figure 18. Therefore, the generated power is higher in terms of the average value with the use of the ANN-based airflow control.

### 5.3. Control Assessment with Real Measured Wave Data

**Figure 19:**
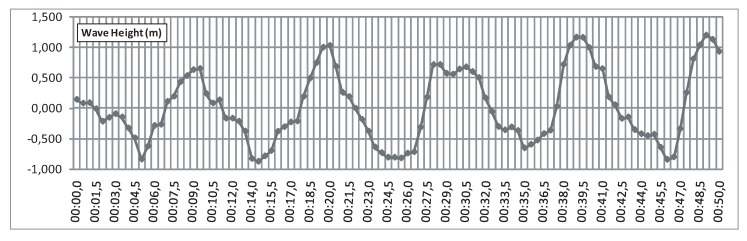
Measured wave input to the implemented OWC model.

In this comparative study, we considered real surface elevation measurements of waves in Mutriku obtained by the Acoustic Doppler Current Profiler on 12 May 2014, at 00:00:00, as shown in Figure 19. This measured input will help test the proposed control with other wave amplitudes and periods.

**Figure 20:**
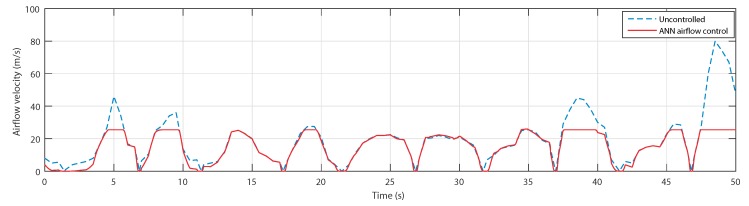
Airflow speed for uncontrolled case and ANN-based airflow control.

The curves of the airflow speed in Figure 20 showed fast airflow at the peak of the waves, namely at 5, 8, 38, and 48 s. Effectively, the ANN controller activated the airflow control, and the speed has been reduced by the air valve.

**Figure 21:**
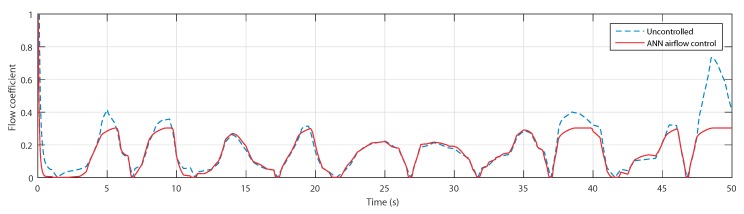
Flow coefficient for uncontrolled case and ANN-based airflow control.

Confirming with the curves of the airflow speed, the flow coefficients, shown in Figure 21, have indeed exceeded the threshold value at the indicated instances when uncontrolled. The controlled OWC, however, shows a regulation of the coefficient at the threshold value 0.3.

**Figure 22:**
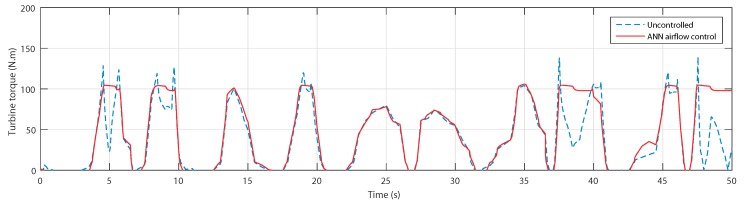
Turbine torque for uncontrolled case and ANN-based airflow control.

The torques shown in Figure 22 prove that the uncontrolled torque did indeed suffer from the stall effect at the same instants, but thanks to the airflow control, they were successfully avoided.

**Figure 23:**
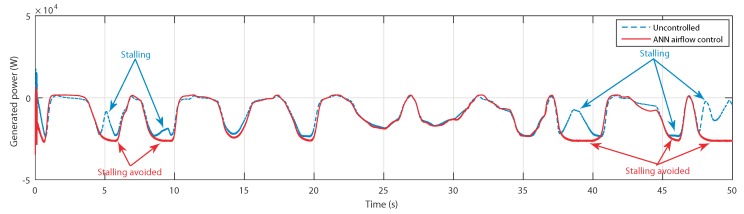
Generated power for uncontrolled case and ANN-based airflow control.

Same as the torques, the generated powers have successfully avoided the stalling effect, as indicated in Figure 23.

**Figure 24:**
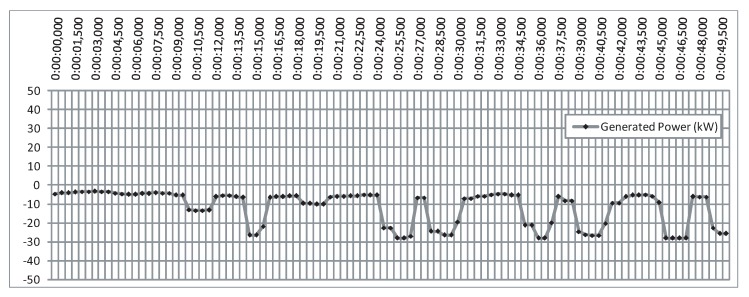
Generated power measured at the NEREIDA wave power plant of Mutriku.

For a final comparison, Figure 24 shows the output power of the OWC measured in the Mutriku facility at the same time and on the same day (12 May 2014, at 00:00:00). These data were provided by the Basque Energy Agency (EVE).

By taking into consideration the quality of the data series due to the large sampling period, one can see the same waveform in both the numerical power of Figure 23 and Figure 24. However, at the instants 5, 8, 18, 38, and 48 s, the controlled power is superior to the measured power.

## 6. Conclusions

In this article, the modeling and implementation of an OWC system using a Wells turbine coupled to a DFIG has been presented. The paper also proposes a novel ANN-based airflow control for the OWC to deal with the stalling behavior of the Wells turbine, which proves to limit the generated power.

The proposed ANN-airflow control uses a trained artificial neural network to recognize waves with high airflow speed that may cause a stalling effect in the system. The data used to train the ANN were gathered by an Acoustic Doppler Current Profiler. The ANN is then able to generate an adequate airflow speed reference. The reference is used in the airflow control scheme. To design the suitable network structure for the problem, numerous simulations were performed using the trial-and-error rule based on the forward approach procedure. The best topology has been selected by considering the best performance index MSE, the number of epochs, and the least complicated structure.

The results of the training process showed that the best MSE is obtained with deep neural networks. The considered ANN topology was able to converge in an acceptable number of epochs. Using the trained network in the airflow scheme, the airflow strategy has been able to detect strong waves and avoid the stalling behavior. The results prove the effectiveness of the control with different sea states. Also, a comparative study using real surface elevation measurements of waves in Mutriku showed good avoidance of the stalling phenomenon even compared to the measured power output of the NEREIDA plant in Mutriku.

## Figures and Tables

**Table 1:** Plant parameters from the NEREIDA wave power plant at Mutriku.

Capture Chamber	Wells Turbine	DFIG Generator	
$w_{c}$ = 4.5 m	*n* = 5	$R_{s}$ = 0.5968 Ω	$P_{rated}$ = 18.45 kW
$l_{c}$ = 4.3 m	*b* = 0.21 m	$R_{r}$ = 0.6258 Ω	$Vs_{rated}$ = 400 V
$\rho_{a}$ = 1.19 ${\mathrm{kg}/m}^{3}$	*l* = 0.165 m	$L_{ss}$ = 0.0003495 H	$f_{rated}$ = 50 Hz
$\rho_{w}$ = 1029 ${\mathrm{kg}/m}^{3}$	*r* = 0.375 m	$L_{rr}$ = 0.324 H	p=2
	*a* = 0.4417 $m^{2}$	$L_{m}$ = 0.324 H	

**Table 2:** Training performance of different structures using the Levenberg–Marquardt algorithm.

Network Structure	Epochs	MSE	Network Structure	Epochs	MSE
	11	1.1196		237	1.0463 × 10${}^{-3}$
2 × 2 × 1	20	8.0244 × 10${}^{-1}$	2 × 2 × 2 × 1	453	9.8214 × 10${}^{-4}$
	56	7.3278 × 10${}^{-1}$		619	8.1576 × 10${}^{-4}$
	98	3.8778 × 10${}^{-1}$		862	8.6248 × 10${}^{-4}$
	68	1.5626 × 10${}^{-1}$		202	8.5032 × 10${}^{-4}$
2 × 4 × 1	122	9.0921 × 10${}^{-2}$	2 × 4 × 2 × 1	534	7.9368 × 10${}^{-4}$
	566	6.6619 × 10${}^{-2}$		811	8.2294 × 10${}^{-4}$
	698	4.2215 × 10${}^{-2}$		1000	7.6146 × 10${}^{-4}$
	188	4.2318 × 10${}^{-2}$		115	9.0408 × 10${}^{-4}$
2 × 8 × 1	528	5.4049 × 10${}^{-3}$	2 × 2 × 4 × 1	482	7.7924 × 10${}^{-4}$
	706	7.6852 × 10${}^{-3}$	(ANN1)	726	8.1396 × 10${}^{-4}$
	1000	2.3425 × 10${}^{-3}$		1000	6.4947 × 10${}^{-4}$
	307	1.7154 × 10${}^{-3}$		98	8.3164 × 10${}^{-4}$
2 × 16 × 1	423	1.3220 × 10${}^{-3}$	2 × 4 × 4 × 1	137	6.9595 × 10${}^{-5}$
	775	7.3013 × 10${}^{-4}$	(ANN2)	546	8.0274 × 10${}^{-4}$
	1000	8.8384 × 10${}^{-4}$		934	7.0812 × 10${}^{-4}$

## Nomenclature

The following symbols are used in this manuscript:

λ,A,H	Wavelength, amplitude, and height (*m*)
h,z	Sea depth and wave surface elevation (*m*)
$T_{w},\omega$	Wave period (s) and wave frequency (rad/s)
*g*	Acceleration gravity ($m/s^{2}$)
P,dP	Capture chamber pressure and Pressure drop (Pa)
$w_{c},l_{c}$	Capture chamber inner width and length (*m*)
V,Q	Capture chamber volume ($m^{3}$) and flow rate ($m^{3}/s$)
$\rho ,v_{x}$	Atmospheric density ($kg/m^{3}$) and airflow speed (m/s)
l,b,D	Blade chord length, blade span and turbine diameter (*m*)
n,p,k,K	Blade number, pole number, wave number and turbine constant
$T_{e},T_{t}$	Electromagnetic and turbine torques (N.m)
*J*	Turbo-generator inertia ($kg.m^{2}$)
$C_{t},C_{a},\phi$	Torque, power and flow coefficients
$R_{s},R_{r}$	Stator and rotor resistances (Ω)
$L_{s},L_{r}$	Stator and rotor inductances (*H*)
$i_{s},i_{r}$	Stator and rotor currents (*A*)
$\psi_{s},\psi_{r}$	Stator and rotor flux (Wb)
$\omega_{s},\omega_{r}$	Stator and rotor rotational speed (rad/s)
$S_{j},\varphi_{j},b_{j}$	Sum function, activation function, bias and output of the $j^{th}$ neuron
$y_{i},y_{j}$	output of $i^{th}$ neuron from previous layer and output of the $j^{th}$ neuron from current layer
$w_{ji}$	Weight of signal connecting $i^{th}$ neuron from previous layer to $j^{th}$ neuron of current layer
